# Cognitive trajectories preluding the imminent onset of Alzheimer’s disease dementia in individuals with normal cognition: results from the HELIAD cohort

**DOI:** 10.1007/s40520-022-02265-y

**Published:** 2022-11-02

**Authors:** Ioannis Liampas, Vasileios Siokas, Eva Ntanasi, Mary H. Kosmidis, Mary Yannakoulia, Paraskevi Sakka, Georgios M. Hadjigeorgiou, Nikolaos Scarmeas, Efthimios Dardiotis

**Affiliations:** 1 Department of Neurology, University Hospital of Larissa, School of Medicine, University of Thessaly, Mezourlo Hill, 41100 Larissa, Greece; 2 1St Department of Neurology, Aiginition Hospital, National and Kapodistrian University of Athens Medical School, Athens, Greece; 3 Department of Nutrition and Dietetics, Harokopio University, Athens, Greece; 4 Laboratory of Cognitive Neuroscience, School of Psychology, Aristotle University of Thessaloniki, Thessaloniki, Greece; 5 Athens Association of Alzheimer’s Disease and Related Disorders, Marousi, Athens, Greece; 6 Department of Neurology, Medical School, University of Cyprus, Nicosia, Cyprus; 7 Taub Institute for Research in Alzheimer’s Disease and the Aging Brain, The Gertrude H. Sergievsky Center, Department of Neurology, Columbia University, Columbia, NY USA

**Keywords:** Attention, Executive function, Language, Memory, Visuospatial ability, Mild cognitive impairment

## Abstract

**Background:**

The cognitive trajectories of cognitively normal (CN) individuals rapidly progressing to Alzheimer’s disease dementia (AD) have not been investigated.

**Aim:**

To explore the preclinical pattern of cognitive performance heralding the rapid progression from normal cognition to AD.

**Methods:**

The HELIAD cohort underwent comprehensive neuropsychological assessments (memory, language, attention, executive and visuo-perceptual functions) at baseline and after approximately 3-year intervals. The cognitive trajectories of those with normal cognition at baseline were explored according to the follow-up diagnosis using adjusted generalised estimating equations analyses.

**Results:**

A total of 932 predominantly female (61%), older (72.9 ± 4.9), CN participants were followed for 3.09 (± 0.83) years. Among them, 761 individuals remained CN, 29 progressed to AD and 142 developed MCI (33 single-domain amnestic, 41 multidomain amnestic, 37 single-domain non-amnestic and 31 multidomain non-amnestic). Those progressing to AD were already performing worse than the healthy reference in every single cognitive domain at baseline. Cognitive deficits ranged between ~ 0.5SD (attention, executive function and language) and ~ 1.0SD (memory and visuo-perceptual skills). Throughout the 3-year follow-up, memory constantly exhibited the most prominent impairment compared to the remaining cognitive domains while executive function diminished in the most abrupt fashion (~ 0.19SD yearly) separating from the remaining three cognitive functions before the development of full-blown AD. Heterogeneous patterns of cognitive decline clearly differentiated those progressing to MCI from those rapidly converting to AD, as well.

**Discussion:**

Poor performance in every cognitive domain may characterise cognitively normal individuals at high risk of fast progression to AD.

**Conclusion:**

Strict neuropsychological cut-offs fail to detect a considerable number of individuals at high risk of rapid progression to AD.

**Supplementary Information:**

The online version contains supplementary material available at 10.1007/s40520-022-02265-y.

## Introduction

The identification of biomarkers which may assist in the preclinical detection of individuals under an increased risk of developing dementia could contribute to the early implementation of interventions that may delay or prevent cognitive impairment (or serve relevant research purposes). Previous studies have consistently indicated the value of several imaging and laboratory indices which can reveal undergoing neuropathological alterations in a prodromal stage [1, 2]. However, increasing interest is accumulating towards the discovery of clinical markers that may partially replace these indices or serve initial screening purposes prior to the implementation of more costly and interventional procedures.

To that end, several researchers have reported that memory complaints and cognitive changes may start even as early as 15 years before the clinical identification of Alzheimer’s Disease (AD) [3, 4]. However, most studies agree that the majority of those who will eventually develop AD tend to undergo an accelerated course of cognitive decline during the last 3 to 8 years prior to the formal diagnoses of both entities (compared to those who do not develop cognitive impairment) [5, 6]. In specific, episodic memory deficits usually manifest earlier, about 7 to 8 before the onset of the disorder, while language, visuo-perceptual skills, executive function and attention tend to ensue, approximately 3 to 4 years before the identification of AD [7–10].

Advancing along the clinical continuum towards AD, usually involves the key intermediate stage of mild cognitive impairment (MCI) due to AD [11]. The speed of progression from normal cognition to MCI and subsequently AD may vary significantly among cognitively normal (CN) individuals while the process of cognitive decline occasionally violates the traditional clinical staging nomenclature, with a number of individuals progressing to AD without a transitional MCI conversion. Previous studies investigating the preclinical cognitive trajectories of CN individuals towards AD did not, however, account for the potential discrepancies between those rapidly progressing to AD (with a relatively short-lasting transition or without a transitional development of MCI) and those exhibiting a slower course of cognitive decline.

In view of the aforementioned literature gap, we decided to undertake the present study. Our objective was to compare the 3-year—preclinical cognitive trajectories of CN individuals rapidly progressing to AD in relation to the trajectories of those progressing to MCI or maintaining normal cognition (as the healthy reference). In this way, we aimed to delineate potential discrepancies in the preclinical patterns of cognitive performance that may serve as prognostic markers for the imminent development of AD in individuals with normal cognition. For this purpose, we capitalized on data from the older, population-based HELIAD (Hellenic Investigation of Aging and Diet) cohort.

## Methods

### Study design, participants and settings

Our sample was derived from the HELIAD study, the rationale and key elements of which have been previously described in great detail [12, 13]. In brief, the HELIAD is a multidisciplinary, population-based, prospective cohort exploring the descriptive and analytical epidemiology of dementia and cognitive impairment in the older Greek population. The Institutional Ethics Review Boards of the University of Thessaly (138/0-07-2009) and the National and Kapodistrian University of Athens (256/10-05-2021) approved all procedures prior to the initiation of the study. Informed consent was acquired from all participants or surrogates prior to participation.

Older (≥ 65 years) participants were randomly selected from the registries of two Greek municipalities, Larissa (an urban–rural area in the province of Thessaly) and Marousi (a suburban area in the metropolitan city of Athens). Collaborative assessments (2–2.5 h long) were conducted by certified neurologists, trained neuropsychologists, and dieticians during baseline and follow-up (approximately after 3-year intervals). A maximum of two assessments (baseline and 2nd visit) are readily available per individual, so far. Relevant information was collected from participants or participant carers (first-degree relatives, etc.), whenever deemed necessary. For the present analysis, eligible individuals were CN at baseline and had available follow-up assessments.

### Diagnostic procedures

Dementia and possible-probable AD were diagnosed using the Diagnostic and Statistical Manual of Mental Disorders -IV-text revision criteria and the National Institute of Neurological and Communicative Disorders and Stroke/Alzheimer Disease and Related Disorders Association criteria correspondingly [14, 15]. Mild cognitive impairment and its subtypes were diagnosed according to the Petersen criteria [11]. MCI was classified as single-domain, non-amnestic (naMCI-SD) in case of isolated language, attention-speed, executive or visuo-perceptual impairment and as multidomain, non-amnestic (naMCI-MD) in case of any combination of the above-listed impairments (not involving episodic memory). MCI was classified as single-domain, amnestic (aMCI-SD) in case of isolated memory impairment and as multidomain, amnestic (aMCI-MD) in case of any combination of cognitive impairment involving episodic memory (i.e., with language and/or attention-speed and/or executive function and/or visuo-perceptual function). Cognitive diagnoses were established during expert consensus meetings, involving a consortium of senior neurologists (E.D., G.M.H. and N.S.) and neuropsychologists (M.H.K.). A detailed description of the diagnostic approach is provided elsewhere [16, 17].

### Neuropsychological assessments

A comprehensive neuropsychological evaluation was performed by trained neuropsychologists: Global cognition and Orientation (MMSE) [18], Non-verbal and Verbal Memory (Medical College of Georgia—MCG—Complex Figure Test [19]; Greek Verbal Learning Test [20]), Language (semantic and phonological verbal fluency [21]; subtests of the Greek version of the Boston Diagnostic Aphasia Examination short form, namely, the Boston Naming Test-short form, and selected items from the Complex Ideational Material Subtest, to assess verbal comprehension and repetition of words and phrases [22]), Visuospatial Ability (Judgment of Line Orientation [23, 24] abbreviated form; MCG Complex Figure Test copy condition, Clock Drawing Test [25]), Attention and Information Processing Speed (Trail Making Test—TMT— [26] Part A), Executive Functioning (TMT Part B; Anomalous Sentence Repetition; Graphical Sequence Test; Motor Programming [19]; months forwards and backwards), and a gross estimate of Intellectual level (a Greek multiple choice vocabulary test [27]) were administered.

Raw scores from each individual neuropsychological test were converted into z-scores using mean and standard deviation values, estimated from the cognitively normal group of individuals at baseline (no dementia or mild cognitive impairment). Subsequently, z-scores of individual tests were averaged to produce domain z-scores for memory, language, attention, executive and visuospatial ability, for each participant (grouping was performed according to an a priori neuropsychological knowledge of the particular cognitive functions reflected by each test [28]). These domain z-scores were in turn averaged to calculate a composite cognitive score, reflecting the overall cognitive status of each individual. Higher z-scores were consistent with better cognitive performance.

### Factors and covariates considered

In an effort to isolate the differential effect of the underlying, preclinical, evolving neurocognitive processes characterising MCI and AD, multiple potential confounders were accounted for in our analyses: age at baseline, years of formal schooling, body-mass index (in kg/m^2^), physical activity (in kcal/day, excluding energy expenditure at sleep) and daily energy intake (in kcal/day) were treated as continuous variables [29]; sex (male–female), previous main occupation (manual-mental) [12], socioeconomic status (low–high) [30], depression and anxiety (yes–no) [30], sleep quality (poor-regular-good) [31] and duration (short-regular-long) [32], current smoking (yes–no), medical history of hypertension, diabetes mellitus, dyslipidaemia, cardiovascular, cerebrovascular and peripheral vascular disease were treated as categorical parameters.

Detailed procedures and definitions for the non-vascular confounders have been provided elsewhere and were, therefore, omitted from the current report. Hypertension was diagnosed based on participants’ self-report and/or blood pressure measurements; systolic/diastolic blood pressure values greater than 140/90 mmHg. Diabetes mellitus was diagnosed according to participants’ self-report and/or pharmacotherapy with insulin and/or oral hypoglycaemics. The diagnosis of dyslipidaemia was established based on participants’ self-report and/or use of lipid-lowering agents. A positive history of cardiovascular disease was defined as a positive history of coronary artery disease and/or a myocardial infraction and/or congestive heart failure. A positive history of cerebrovascular disease was defined as a positive history of stroke and/or mini-stroke and/or transient ischemic attack.

### Statistical analysis

Our aim was to delineate the preclinical pattern of cognitive performance preluding the imminent onset of AD in individuals with normal cognition. Baseline differences between the group that converted to AD after the 3-year follow-up and those progressing to MCI or maintaining normal cognition were examined using the independent samples *t*-test (scale variables) and Pearson’s chi-squared test (categorical variables). The longitudinal cognitive trajectories of the CN individuals were explored according to the cognitive diagnosis at follow-up using adjusted generalised estimating equations (GEE) analyses. GEE accounts for the potential correlation of repeated measurements in the same individual. We treated each participants’ baseline and follow-up evaluations as a cluster. Exchangeable (compound symmetry) covariance matrices were conventionally chosen as working correlation structures. Consecutive adjusted GEE models were explored using global and individual domain cognitive indices as the dependent scale variables. Apart from the main effects of the above-listed confounders (previous section), each GEE model featured the main effect of time (in years from baseline), the main effects of the follow-up diagnoses (CN, naMCI-SD, na-MCI-MD, aMCI-SD, aMCI-MD, AD) and the follow-up diagnoses by time interactions (six interactions).

To retain the power of our study despite the inclusion of multiple confounders in our analyses (different participant sets had missing values per parameter), automatic multiple data imputation was conducted for the above-listed potential confounders. Neuropsychological assessments were exclusively used as auxiliary-predictive variables, whereas all confounders were both imputed and used as auxiliary-predictive variables (age, sex, education and socioeconomic status were the only parameters without any missing values). All statistical analyses were performed using the IBM SPSS Statistics Software Version 25 (Chicago, IL, USA). The conventional significance threshold of *α* = 0.05 was set for revealing significant interactions.

## Results

### Participant characteristics and missing data

The HELIAD cohort included 1107 participants who had undergone at least one follow-up neuropsychological assessment. Among them, 28 were diagnosed with dementia at baseline, 118 were diagnosed with MCI while 4 had inconclusive cognitive diagnoses and were excluded from the present investigation. From the remaining 957 participants, 5 were diagnosed with dementia other than AD at follow-up while another 20 had missing data leading to inconclusive cognitive diagnoses and were excluded from our analysis. A total of 932 CN, predominantly female (61%), older (72.9 ± 4.9) participants were finally included in the current report. After a mean follow-up of 3.09 (± 0.83) years, 761 subjects remained unimpaired, 29 progressed to AD, whereas 142 converted to MCI. Among them, 33 were diagnosed with aMCI-SD, 41 with aMCI-MD, 37 with naMCI-SD and 31 with naMCI-MD. The baseline characteristics of the CN baseline sample according to the follow-up diagnoses are presented in Table 1. Differences between the group that progressed to AD and the rest of the participants are provided. Participants that progressed to AD were older and less educated than those with normal cognition at follow-up. Vascular comorbidities and sleep disorders were equally prevalent between those who developed AD and the rest of the participants, whereas the frequency of depression was found elevated in those converting to AD compared to those remaining unimpaired. Of note, individuals who progressed to AD performed worse in episodic memory compared to both those progressing to MCI and those remaining unimpaired, whereas regarding attention only the latter group outperformed those that converted to AD. Throughout the rest of the Results section, the participant groups will be discriminated using their follow-up cognitive diagnoses: for example, the ‘‘AD group’’ or ‘‘the group that progressed to AD’’ will both refer to those who were diagnosed with AD at follow-up. All results will be reported in units of standard deviation (SD). Differences in baseline cognitive performance and rates of decline will be reported relative to the baseline performance and decline rates of the healthy reference (CN group).

**Table 1:** Baseline participant characteristics according to the follow-up cognitive diagnosis

Parameter	AD (*n* = 29)	naMCI-MD (*n* = 31)	naMCI-SD (*n* = 37)	aMCI-MD (*n* = 41)	aMCI-SD (*n* = 33)	CN (*n* = 761)	Total (*n* = 932)
Age at baseline (*n* = 932)	77.0 ± 4.9	74.8 ± 5.0	72.8 ± 4.9*	75.2 ± 5.7	74.0 ± 5.2*	72.5 ± 4.7*	72.9 ± 4.9
Years of education (*n* = 932)	6.6 ± 4.8	4.8 ± 3.6	7.4 ± 4.7	6.1 ± 4.4	9.4 ± 4.6*	8.7 ± 4.9*	8.4 ± 4.9
Sex (male%) (*n* = 932)	13 (49%)	11 (35%)	8 (22%)*	18 (44%)	19 (58%)	297 (39%)	366 (39%)
Main occupation (mental%) (*n* = 837)	8 (29%)	1 (3%)*	11 (34%)	9 (24%)	17(63%)*	269 (39%)	315 (38%)
Socioeconomic status (lower%) (*n* = 932)	14 (48%)	18 (58%)	16 (43%)	19 (46%)	17 (52%)	313 (41%)	397 (43%)
Body-mass index kg/m^2^ (*n* = 918)	28.7 ± 3.7	28.3 ± 3.6	28.9 ± 4.6	28.8 ± 3.7	28.3 ± 3.7	28.9 ± 4.5	28.9 ± 4.4
Physical activity (kcal/day) (*n* = 920)	2254 ± 345	2248 ± 308	2403 ± 580	2305 ± 399	2313 ± 336	2378 ± 481	2366 ± 470
Energy intake (kcal/day) (*n* = 904)	1974 ± 549	2180 ± 595	1898 ± 623	2042 ± 597	1903 ± 473	1993 ± 532	1993 ± 539
Depression (Yes%) (*n* = 931)	9 (31%)	5 (16%)	14 (38%)	4 (10%)*	11 (33%)	116(15%)*	159 (17%)
Anxiety (Yes%) (*n* = 931)	5 (17%)	4 (13%)	1 (3%)*	4 (10%)	3 (9%)	87 (11%)	104 (11%)
Sleep quality (poor-regular-good%) (*n* = 906)	8/8/11 (30/30/40%)	11/8/12 (35/26/39%)	15/12/10 (41/32/27%)	13/18/9 (33/45/ 22%)	9/14/10 (27/43/30%)	214/221/303 (29/30/31%)	270/281/355 (30 /31 /39%)
Sleep duration (short-regular-long%) (*n* = 926)	9/18/2 (31/62/7%)	10/19/1 (33/64/3%)	6/28/2 (17/77/ 6%)	12/28/1 (29/68/3%)	6/26/1 (18/79/3%)	184/525/48 (24/70/6%)	227/644/55 (25/70/5%)
Current smoking (Yes%) (*n* = 915)	3 (10%)	1 (3%)	2 (6%)	3 (7%)	2 (7%)	93 (12%)	104 (11%)
Hypertension (Yes%) (*n* = 924)	17 (59%)	23 (74%)	23 (64%)	30 (73%)	23 (74%)	551 (73%)	667 (72%)
Diabetes mellitus (Yes%) (*n* = 915)	5 (17%)	9 (31%)	8 (22%)	8 (20)	3 (10%)	126 (17%)	159 (17%)
Dyslipidaemia (Yes%) (*n* = 916)	15 (52%)	16 (52%)	22 (63%)	17 (41%)	17 (59%)	365 (49%)	452 (49%)
Cardiovascular disease (Yes%) (*n* = 915)	4 (14%)	2 (6%)	4 (11%)	4 (10%)	2 (7%)	85 (11%)	101 (11%)
Cerebrovascular disease (Yes%) (*n* = 917)	0 (0%)	3 (10%)	3 (8%)	2 (5%)	2 (7%)	48 (6%)	58 (6%)
Peripheral vascular disease (Yes%) (*n* = 916)	1 (3%)	2 (6%)	1 (3%)	0 (0%)	1 (3%)	20 (3%)	25 (3%)
Global cognition(*n* = 921)	−0.96 ± 0.96	−0.58 ± 0.57	−0.14 ± 0.60*	−0.57 ± 0.67	0.02 ± 0.54*	0.12 ± 0.62*	0.02 ± 0.68
Episodic memory (*n* = 916)	−1.12 ± 0.75	−0.47 ± 0.72*	−0.04 ± 0.83*	−0.72 ± 0.75*	−0.25 ± 0.62*	0.19 ± 0.79*	0.06 ± 0.84
Language (*n* = 920)	−0.77 ± 1.05	−0.46 ± 0.69	−0.10 ± 0.67*	−0.53 ± 0.92	−0.32 ± 0.55*	0.16 ± 0.72*	0.07 ± 0.77
Executive function (*n* = 917)	−0.72 ± 0.74	−0.62 ± 0.67	−0.10 ± 0.58*	−0.61 ± 0.84	0.06 ± 0.47*	0.11 ± 0.65*	0.02 ± 0.69
Visuospatial ability (*n* = 910)	−1.07 ± 1.81	−0.38 ± 0.92	−0.16 ± 0.69*	−0.37 ± 0.92*	0.08 ± 0.79*	0.11 ± 0.73*	0.03 ± 0.83
Attention-speed (*n* = 883)	−0.61 ± 0.95	−0.94 ± 1.31	−0.30 ± 0.91	−0.44 ± 0.90	−0.09 ± 1.30	0.07 ± 0.90*	−0.02 ± 0.96

*AD* Alzheimer’s disease, *MCI* mild cognitive impairment, *aMCI-MD* multidomain amnestic MCI, *aMCI-SD* single-domain amnestic MCI, *naMCI-MD* multidomain non-amnestic MCI, *naMCI-SD* single-domain non-amnestic MCI, *CN* cognitively normal

*Denotes a significant difference (*p* < 0.05) between the CN or MCI groups and the AD group according to the independent samples *t*-test (scale variables) or the Pearson’s chi-squared test (categorical variables)

### The preclinical pattern of cognitive performance heralding the direct onset of AD

Tables 2 and 3 summarize the predicted baseline differences and annual rates of change in the 3-year—preclinical cognitive trajectories according to the cognitive diagnosis at follow-up. In general, those who progressed to AD corresponded to the pathological extreme of our sample in terms of global cognition performing worse at baseline and declining in a more precipitous manner during the 3-year follow-up. Of note, the 3-year, preclinical pattern of cognitive performance differentiated quite well between the group that converted to AD and the rest of the participants.

**Table 2:** Adjusted regression coefficients of cognitively normal (CN) older adults (> 64 years), according to the generalized estimating equations (GEE) analysis

Baseline differences and annual rates of change	Global cognition	Episodic memory	Language	Executive function	Visuospatial skills	Attention-speed
AD	−**0.75 (**−**1.01, **−**0.49), < 0.001**	−**0.98 (**−**1.22, **−**0.74), < 0.001**	−**0.59 (**−**0.85, **−**0.33), < .001**	−**0.58 (**−**0.80, **−**0.37), < 0.001**	−**0.96 (**−**1.57, **−**0.35), 0.002**	−**0.47 (**−**0.86, **−**0.08), 0.019**
AD*time	−**0.15 (**−**0.21, **−**0.08), < 0.001**	−**0.17 (**−**0.26, **−**0.08), < 0.001**	−**0.17 (**−**0.25, **−**0.09), < 0.001**	−**0.22 (**−**0.30, **−**0.13), < 0.001**	0.03 (−0.15, 0.21), 0.732	−0.19 (−0.45, 0.07), 0.146
naMCI−MD	−**0.33 (**−**0.53, **−**0.14), 0.001**	−**0.34 (**−**0.58, **−**0.11), 0.005**	−0.19 (−0.42, 0.04), 0.098	−**0.36 (**−**0.59, **−**0.14), 0.002**	−0.17 (−0.48, 0.14), 0.275	−**0.59 (**−**1.10, **−**0.08), 0.023**
naMCI-MD*time	−**0.06 (**−**0.10, **−**0.01), 0.011**	−0.06 (−0.13, 0.01), 0.092	−**0.10 (**−**0.15, **−**0.04), < 0.001**	−0.03 (−0.09, 0.04), 0.435	−0.05 (−0.12, 0.02), 0.143	−0.08 (−0.24, 0.08), 0.320
naMCI-SD	−**0.13 (**−**0.23, **−**0.02), 0.016**	−0.17 (−0.36, 0.01), 0.065	−0.11 (−0.26, 0.03), 0.132	−0.09 (−0.23, 0.06), 0.243	−0.09 (−0.25, 0.08), 0.286	−0.20 (−0.42, 0.02), 0.078
naMCI-SD*time	−**0.05 (**−**0.08, **−**0.02), 0.003**	−0.04 (−0.09, 0.02), 0.217	−**0.07 (**−**0.12, **−**0.01), 0.020**	−**0.05 (**−**0.09, **−**0.00), 0.033**	−0.05 (−0.12, 0.01), 0.100	−0.07 (−0.15, 0.02), 0.111
aMCI-MD	−**0.39 (**−**0.55, **−**0.23), < 0.001**	−**0.61 (**−**0.83, **−**0.38), < 0.001**	−**0.34 (**−**0.55, **−**0.14), 0.001**	−**0.45 (**−**0.66, **−**0.25), < 0.001**	−**0.27 (**−**0.50, **−**0.04), 0.024**	−0.22 (−0.57, 0.12), 0.202
aMCI-MD*time	−**0.12 (**−**0.18, **−**0.06), < 0.001**	−**0.18 (**−**0.24, **−**0.12), < 0.001**	−**0.12 (**−**0.21, **−**0.04), 0.003**	−0.06 (−0.13, 0.01), 0.114	−**0.16 (**−**0.26, **−**0.05), 0.003**	−0.03 (−0.16, 0.10), 0.691
aMCI-SD	−0.09 (−0.23, 0.04), 0.180	−**0.43 (**−**0.62, **−**0.24), < 0.001**	0.13 (−0.01, 0.26), 0.064	−0.08 (−0.22, 0.06), 0.269	−0.01 (−0.24, 0.22), 0.918	−0.07 (−0.44, 0.30), 0.707
aMCI-SD*time	−0.03 (−0.06, 0.00), 0.070	−**0.12 (**−**0.18, **−**0.06), < 0.001**	−0.01 (−0.05, 0.03), 0.575	−0.01 (−0.04, 0.02), 0.615	−0.02 (−0.08, 0.05), 0.596	0.01 (−0.07, 0.09), 0.800
CN	Ref	Ref	Ref	Ref	Ref	Ref
CN*time	Ref	Ref	Ref	Ref	Ref	Ref
Main time effect	−**0.07 (**−**0.08, **−**0.06), < 0.001**	−**0.05 (**−**0.07, **−**0.04), < 0.001**	−**0.06 (**−**0.07, **−**0.05), < 0.001**	−**0.05 (**−**0.06, **−**0.04), < 0.001**	−**0.11 (**−**0.12, **−**0.09), < 0.001**	−**0.06 (**−**0.08, **−**0.04), < 0.001**

Those who remained CN at follow-up were used as the healthy reference

Bold denotes statistical significance

*AD* Alzheimer’s disease, *MCI* mild cognitive impairment, *aMCI-MD* multidomain amnestic MCI, *aMCI-SD* single-domain amnestic MCI, *naMCI-MD* multidomain non-amnestic MCI, *naMCI-SD* single-domain non-amnestic MCI, *CN* cognitively normal, *Ref* reference

**Table 3:** Adjusted regression coefficients of cognitively normal (CN) older adults (> 64 years), according to the generalized estimating equations (GEE) analysis

Baseline differences and annual rates of change	Global cognition	Episodic memory	Language	Executive function	Visuospatial skills	Attention-speed
naMCI-MD	**0.42 (0.10, 0.74), 0.011**	**0.64 (0.31, 0.97), < 0.001**	**0.39 (0.05, 0.73), 0.023**	0.22 (−0.09, 0.52), 0.159	**0.79 (0.10, 1.48), 0.025**	−0.12 (−0.76, 0.52), 0.711
naMCI-MD*time	**0.09 (0.01, 0.17), 0.031**	0.11 (−0.00, 0.22), 0.051	0.07 (−0.03, 0.17), 0.157	**0.19 (0.09, 0.30), < 0.001**	−0.09 (−0.27, 0.11), 0.386	0.11 (−0.19, 0.42), 0.464
naMCI-SD	**0.62 (0.34, 0.90), < 0.001**	**0.81 (0.52, 1.11), < 0.001**	**0.48 (0.18, 0.77), 0.002**	**0.50 (0.25, 0.75), < 001**	**0.87 (0.22, 1.52), 0.008**	0.27 (−0.17, 0.71), 0.232
naMCI-SD*time	**0.10 (0.02, 0.17), 0.012**	**0.13 (0.03, 0.24), 0.009**	**0.10 (0.01, 0.20), 0.040**	**0.17 (0.08, 0.26), < 0.001**	−0.09 (−0.27, 0.10), 0.374	0.12 (−0.15, 0.40), 0.368
aMCI-MD	**0.36 (0.06, 0.66), 0.018**	**0.38 (0.06, 0.69), 0.020**	0.25 (−0.08. 0.57), 0.141	0.13 (−0.16, 0.42), 0.386	**0.69 (0.04, 1.35), 0.039**	0.24 (−0.27, 0.76), 0.351
aMCI-MD*time	0.03 (−0.06, 012), 0.512	−0.01 (−0.11, 0.10), 0.887	0.04 (−0.07. 0.16), 0.461	**0.16 (0.05, 0.27), 0.004**	−0.19 (−0.39, 0.02), 0.073	0.17 (−0.12, 0.46), 0.260
aMCI-SD	**0.66 (0.37, 0.95), < 0.001**	**0.55 (0.26, 0.85), < 0.001**	**0.71 (0.43, 1.00), < 0.001**	**0.51 (0.26, 0.75), < 0.001**	**0.96 (0.35, 1.57), 0.005**	0.40 (−0.15, 0.94), 0.155
aMCI-SD*time	**0.12 (**−**0.05, 0.19), 0.001**	0.05 (−0.06, 0.15), 0.361	**0.16 (0.07, 0.25), 0.001**	**0.21 (0.12, 0.30), < 0.001**	−0.05 (−0.24, 0.14), 0.614	0.20 (−0.07, 0.47), 0.141
AD	Ref	Ref	Ref	Ref	Ref	Ref
AD by time	Ref	Ref	Ref	Ref	Ref	Ref

Those who developed AD at follow-up were used as the pathological reference

Bold denotes statistical significance

*AD* Alzheimer’s disease, *MCI* mild cognitive impairment, *aMCI-MD* multidomain amnestic MCI, *aMCI-SD* single-domain amnestic MCI, *naMCI-MD* multidomain non-amnestic MCI, *naMCI-SD* single-domain non-amnestic MCI, *CN* cognitively normal, *Ref* reference

**Fig. 1:**
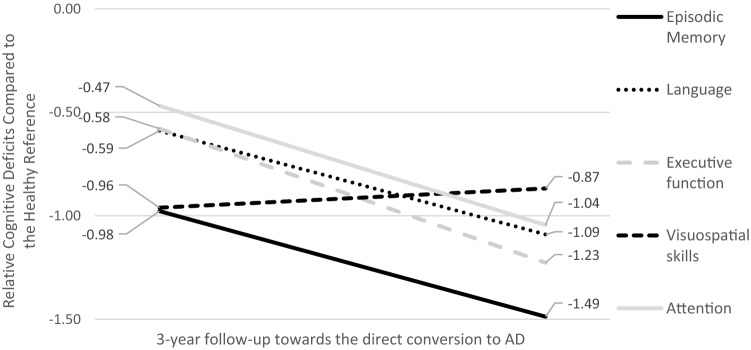
Predicted, 3-year—preclinical pattern of cognitive performance for individuals with normal cognition rapidly progressing to Alzheimer’s disease dementia (AD)

CN participants that developed AD after the 3-year follow-up were already performing worse than the healthy reference in every single cognitive domain at baseline. Relative differences ranged between ~ 0.5 SD (in the domains of attention, executive function and language) and ~ 1.0 SD (in the domains of episodic memory and visuo-perceptual skills). During the 3-year follow-up, with the exception of visuospatial skills (which already exhibited a very prominent decline), the remaining cognitive functions declined in an accelerated fashion (only 12 of the participants that developed AD had available attention assessments at follow-up, hence, the low precision of the relevant estimation). From a qualitative point of view, episodic memory constantly exhibited the worst relative trajectory throughout the 3-year follow-up (the most prominent deficits compared to the other functions), whereas executive function diminished in the most abrupt manner separating from the remaining three cognitive domains before the development of full-blown AD (Fig. 1).

**Fig. 2:**
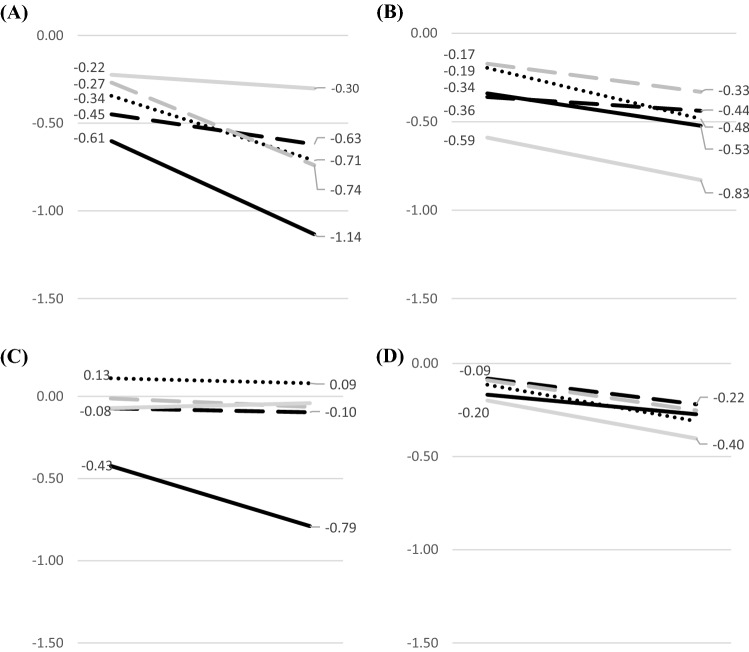
Predicted, 3-year—preclinical patterns of cognitive performance for individuals with normal cognition at baseline progressing to mild cognitive impairment (MCI) at follow-up. The *x*-axis corresponds to the 3-year (mean) follow-up of our sample and the *y*-axis reflects the relative cognitive deficits of each MCI group compared to the healthy reference. Each cognitive domain is represented by a different colour: Episodic Memory—black, regular line; Language—black, dotted line; Executive Function—black, broken line; Visuo-spatial Ability—grey, broken line; and Attention—grey, regular line. Four relevant demonstrations are provided: progressing to **A** multidomain, amnestic MCI, **B** multidomain non-amnestic MCI, **C** single-domain amnestic MCI and **D** single-domain non-amnestic MCI

The 3-year, preclinical motifs of cognitive impairment differentiated those converting to AD from those progressing to MCI, as well. Heterogeneous patterns of less prominent cognitive deficits not-involving every single cognitive domain and associated with relatively attenuated annual rates of decline characterized those who developed MCI at the 3-year follow-up. Regarding the multidomain MCI groups in specific, the domain of attention remained relatively intact in the aMCI-MD group (clearly differentiating the aMCI-MD from the AD group), whereas attention deficits were constantly the most prominent in the naMCI-MD group throughout the 3-year follow-up period towards the onset of the disorder (as opposed to the prominent impairment of episodic memory in the AD group) (Fig. 2). Of interest, an intriguing qualitatively assessed trend seemed to exist between the baseline pattern of CN individuals progressing to AD and the follow-up pattern of CN participants that converted to aMCI-MD. In specific, the predicted pattern of cognitive impairment at the onset of aMCI-MD was quite comparable to the baseline pattern of cognitive impairment of those rapidly progressing to AD (cognitive deficits were severity-wise arranged as follows: attention < executive function < language < visuospatial skills < episodic memory) (Online Resource 1).

## Discussion

The present study explored the 3-year—preclinical cognitive trajectories of CN individuals rapidly converting to AD in relation to the trajectories of those progressing to MCI or maintaining normal cognition (as the healthy reference). The preclinical pattern of cognitive performance differentiated quite well between those that converted to AD and the rest of the participants. Considerable, multidomain baseline deficits involving every domain of cognition and associated with predominant episodic memory impairment throughout the 3-year follow-up portended the imminent development of AD. Less prominent, multidomain baseline deficits with intact attention and predominant episodic memory impairment throughout the 3-year follow-up heralded the onset of aMCI-MD. Multidomain baseline deficits involving attention, with predominant attention impairment throughout the 3-year follow-up were a harbinger of naMCI-MD.

Although the present article is the first to examine the preclinical cognitive trajectories portending the imminent onset of AD, previous researchers have also explored the factors related to rapid cognitive decline and incident dementia. Older age, genetic susceptibility, physical inactivity, lower frequency of leisure activities, worse functional status, poorer psychological welfare, neuropsychiatric symptoms, subjective memory concerns, worse nutritional status and poorer vascular health have all been associated with steeper cognitive decline in CN older adults [33–38]. Of note, similar associations have been generated for patients with MCI and more precipitous courses of cognitive decline [39–41] while a number of imaging and laboratory biomarkers have been added to the arsenal of predictors of rapid cognitive decline [42, 43]. The findings of the current paper could be potentially combined with the aforementioned findings to reveal the subgroup of older individuals with normal cognition bearing the highest risk of imminent progression to dementia.

Of note, the baseline pattern of cognitive performance of those rapidly converting to AD was determined quite similar to the predicted pattern of cognitive impairment at the onset of aMCI-MD (after the completion of the 3-year follow-up) (cognitive deficits were arranged as follows in terms of relative severity: attention < executive function < language < visuospatial skills < episodic memory) (Online Resource 1). This finding is of particular interest considering the high (~ 50%), 3-year conversion rate of aMCI-MD to AD [44]. In specific, our results might imply that the aforementioned pattern of cognitive impairment may be interchangeable between MCI patients and CN individuals sharing a particularly high risk of progressing to AD within 3 years. Therefore, it may provide clinicians and researchers with a unique opportunity to identify high-risk individuals either fulfilling the formal diagnosis of MCI (using proper cut-offs in neuropsychological assessments) or not. The implementation of strict neuropsychological cut-offs to detect individuals at risk of progressing to AD (i.e., the identification of those with MCI) most probably yields a suboptimal negative prognostic value, whereas an ‘‘holistic’’ neuropsychological approach integrating the identification of less prominent, subthreshold multidomain cognitive deficits may uncover additional CN individuals at high-risk of imminent AD development.

Previous research has suggested that cognitive changes tend to precede the diagnosis of AD for many years, potentially reflecting the undergoing neurodegenerative alterations which are present even as long as 20–25 years before its onset [3, 4]. Episodic memory, language, attention, visuo-perceptual and executive skills have all been reported to decline prior to the former identification of AD [10]. However, episodic memory dysfunction usually precedes while visuospatial and language decline tend to ensue [3, 4, 6]. On the other hand, attention and executive function-dependent tasks are affected later in the preclinical course towards the development of AD, typically within the last 3 years before its clinical diagnosis [7, 8, 45]. Considering the aforementioned evidence along with the findings of the current report, it appears that the compromisation of episodic memory, language—visuospatial skills, and finally attention—executive function, constitute successive ‘‘severity points’’ in the cognitive continuum between normal aging and AD. Of interest, a prominent acceleration in executive dysfunction seems to constitute a pivotal turning point ultimately leading to full-blown dementia of the AD type [46].

Episodic memory deficits are also arguably the first to forebode the development of MCI due to AD, usually 4–8 years prior to the formal identification of the disorder [47, 48]. Language and visuospatial impairment similarly ensue, manifesting about 3 years prior to the diagnosis of MCI due to AD, whereas executive deficits have been reported shortly after [47, 48]. As it happens, published evidence is even suggestive that poor episodic memory (more strongly) and semantic fluency are fine predictors of the ~ 6-year progression risk from normal cognition to aMCI [49]. According to the current report, however, these associations are probably driven by the prodromal multi-domain decline of those ultimately developing aMCI-MD, whereas CN subjects that progress to aMCI-SD practically manifest with isolated episodic memory deficits up to the formal identification of the disorder.

Unlike the aMCI subgroup, the longitudinal cognitive trajectories towards the development of naMCI have not been investigated to date. The current report has revealed that attention is predominantly affected throughout the 3-year, preclinical course towards the development of naMCI-MD, which is probably consistent with the higher risk of DLB in individuals with naMCI-MD [50]. Of note, minor episodic memory deficits were determined at the beginning of the 3-year follow-up. To that end, previous high-quality research has also provided evidence supporting the relatively early involvement of episodic memory in naMCI-MD. In specific, episodic memory impairment has been reported to confer an increased 6-year risk of naMCI development, whereas semantic fluency has not been determined as such an early indicator of incident naMCI [49].

### Strengths and limitations

The present article compared the 3-year, preclinical cognitive trajectories of CN individuals rapidly converting to AD in relation to the trajectories of those progressing to MCI or maintaining normal cognition. The study sample was drawn from the population-based, prospective HELIAD cohort which consists of a randomly selected sample of older adults from two Greek communities, a provincial and a metropolitan municipality. Participants underwent comprehensive baseline and follow-up neuropsychological evaluation while clinical diagnoses (dementia and MCI) were established during expert-consensus meetings based on standard clinical criteria. All analyses were adjusted for multiple important confounders to isolate the differential effect of the underlying evolving neurodegenerative processes.

However, our study had a number of important limitations, as well. First, although the diagnosis of dementia was clinically established by a consortium of senior experts, it was not supported by imaging and biological biomarkers (potential misclassification bias). Second, our analyses may have been influenced by the loss of follow-up since a non-trivial proportion of our sample did not undergo follow-up investigations. Moreover, despite accounting for the latent impact of a great number of important parameters, our investigations may still be affected by residual confounding. Of note, although the majority of the main vascular determinants of cognitive decline and incident dementia were accounted for, we did not adjust our analyses for the presence of atrial fibrillation, a well-established predictor of cognitive impairment and dementia [51]. Furthermore, apart from AD, the remaining dementia entities were not investigated due to the small number of events per entity (as expected, considering the small prevalence of other dementia entities and the prospective design of the HELIAD study). In addition, the moderate follow-up duration of approximately 3 years, as well as the relatively small number of events at follow-up might have underpowered several investigations. Finally, due to the lack of intermediate assessments, it was infeasible to differentiate CN individuals that directly progressed to AD from those exhibiting a short transitional conversion to MCI before the development of full-blown AD.

## Supplementary Information

Below is the link to the electronic supplementary material.

Supplementary file1 (PDF 431 KB)

## Data Availability

The datasets generated during and/or analysed during the current study are available from the corresponding author [E.D.], upon reasonable request.
