# Supplementary material for: Cognitive trajectories preluding the imminent onset of Alzheimer’s disease dementia in individuals with normal cognition: results from the HELIAD cohort
Source: Aging Clin Exp Res. 2022 Nov 2;35(1):41–51. doi: 10.1007/s40520-022-02265-y (PMC9816286; doi:10.1007/s40520-022-02265-y)

Supplementary Figure 1 Predicted, 3-year - preclinical pattern of cognitive performance for individuals with normal cognition at baseline progressing to multidomain, amnestic mild cognitive impairment (aMCI-MD, left solid lines) vs. baseline pattern of cognitive impairment of individuals with normal cognition and rapid onset of Alzheimer's disease (AD, right solid lines). A qualitative trend is observed between the two groups: the predicted pattern of cognitive impairment at the onset of aMCI-MD is comparable to the baseline pattern of cognitive impairment of those that rapidly converting to AD. The gradient lines were designed to better delineate this qualitative trend

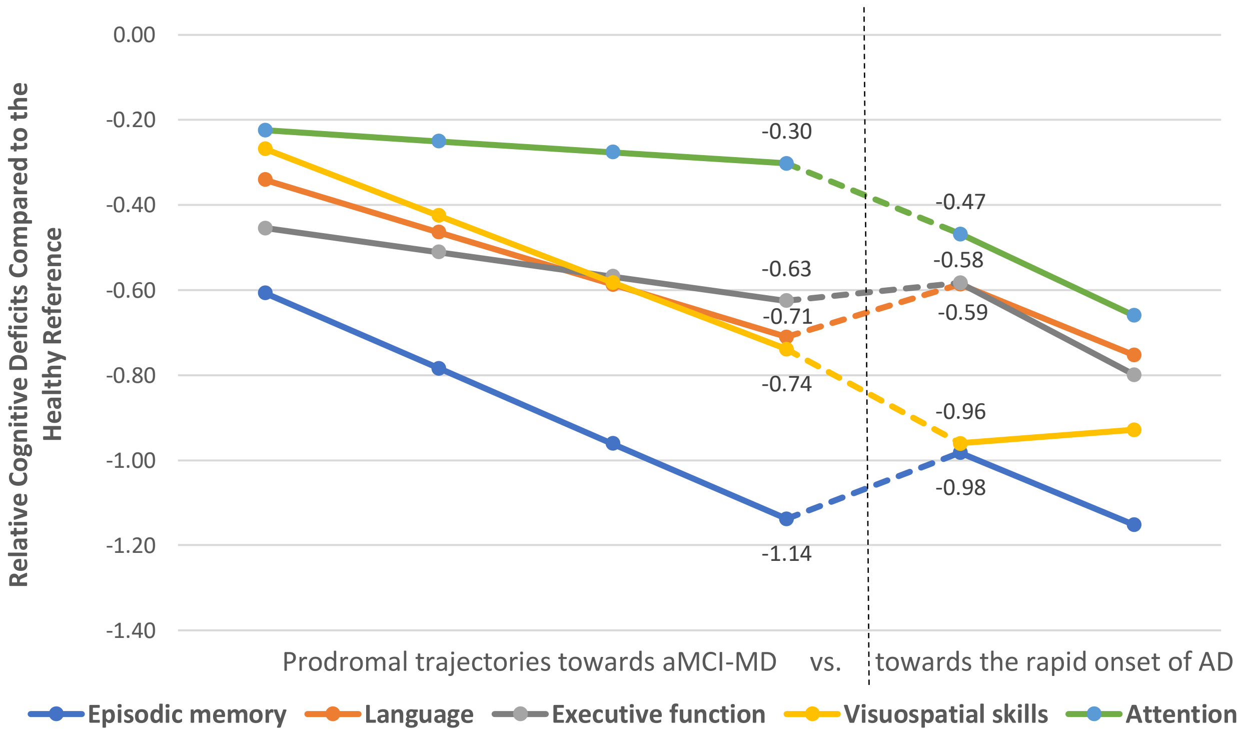

Supplement: Supplementary file 1 — Supplementary file1 (PDF 431 KB) [file 40520_2022_2265_MOESM1_ESM.pdf]
